# Effect of omalizumab for autoimmune progesterone dermatitis refractory to bilateral oophorectomy: a case report

**DOI:** 10.1186/s13223-021-00561-2

**Published:** 2021-06-13

**Authors:** Akshay Varghese, Terri Paul, Harold Kim, Stan Van Uum, Peter Vadas, Alescia Azzola

**Affiliations:** 1 Division of Internal Medicine, Northern Ontario School of Medicine, Sudbury, ON Canada; 2 Division of Endocrinology and Metabolism, Schulich School of Medicine and Dentistry, Western University, London, ON Canada; 3 Division of Clinical Immunology and Allergy, Schulich School of Medicine and Dentistry, Western University, London, ON Canada; 4 Division of Clinical Immunology and Allergy, St. Michael’s Hospital, University of Toronto, Toronto, ON Canada

**Keywords:** Autoimmune progesterone dermatitis, Bilateral oophorectomy, Omalizumab

## Abstract

**Background:**

Autoimmune progesterone dermatitis (APD) is a rare skin condition caused by sensitivity to high levels of progesterone secreted during the luteal phase of the menstrual cycle. This may be due to various pathophysiological mechanisms including a Type I and Type IV hypersensitivity reaction. Here we present the case of a patient with APD whose episodic flares were controlled by the addition of omalizumab, after a bilateral oophorectomy failed to resolve her symptoms.

**Case Presentation:**

A 34-year-old female presented to our Endocrine clinic with marked Cushingoid features secondary to high-dose oral prednisone prescribed for APD diagnosed 6 years earlier. She first developed a pruritic maculopapular rash on her arms and legs just after the birth of her second child in 2009. The rash was also associated with headaches and diffuse angioedema. Symptoms occurred for 1–2 weeks, in a cyclical fashion, during the luteal phase of each menstrual cycle and subsided within a few days after menses. The severity of symptoms increased as time went on, and flare-ups began to also include dyspnea, nausea, vomiting and abdominal pain. Her symptoms improved with administration of oral prednisone, but she continued to experience breakthrough symptoms. After multiple failed treatment modalities, she elected bilateral oophorectomy in 2018. However, her symptoms of APD persisted and she still required high-dose oral prednisone. Her condition was further complicated by vasomotor menopausal symptoms and progressive iatrogenic Cushing’s syndrome. She eventually was started on Omalizumab, which suppressed further recurrences of APD symptoms and allowed her to wean off prednisone. Vasomotor menopausal symptoms responded well to the addition of conjugated estrogens with bazedoxifene. However, her symptoms of diffuse bony pain and arthralgias which started whilst on prednisone have persisted in spite of discontinuing prednisone.

**Conclusions:**

To our knowledge, this is only the third case of APD which was successfully treated with Omalizumab and the first case where a bilateral oophorectomy failed to resolve symptoms of APD in the literature. This case also demonstrates the complications of vasomotor menopausal symptoms secondary to a bilateral oophorectomy, as well as the adverse effects of long-term glucocorticoid therapy.

## Background

Autoimmune progesterone dermatitis (APD) is a rare condition, with a literature review from 2016 documenting approximately 90 cases [1]. Patients with APD are sensitive to the high levels of progesterone secreted during the luteal phase of the menstrual cycle [2]. Depending on whether the reaction is IgE-mediated, or T-cell mediated, APD can present in various forms, such as eczema, folliculitis, and erythema multiforme, but can also progress to more acutely severe manifestations such as dyspnea and anaphylaxis [2]. Symptom onset is typically 1 week prior to menses and symptoms resolve a few days after the onset of menstruation [3, 4].

Although the pathogenesis of the disease is not yet fully understood, it has been postulated to involve Type I and Type IV hypersensitivity reactions [4]. Progesterone has been noted to stimulate immunoglobulin E (IgE)-mediated mast cell degranulation [2, 5]. Additionally, progesterone sensitivity may be also due to cell-mediated immunity, due to prior uptake of progesterone by antigen-presenting cells and stimulation of T-helper cells [4]. Furthermore, cross-reactivity with other endogenous steroid hormones such as 17-α-hydroxy-progesterone may also be a plausible mechanism for increased progesterone sensitivity in APD patients [1].

The diagnosis of APD is based on the unique cyclical appearance of symptoms during a menstrual cycle. If APD is IgE-mediated, the diagnosis is confirmed by a positive wheal and flare response to skin testing with progesterone [6]. Various treatment options have been described for APD, including gonadotrophin-releasing hormone (GnRH) agonists (suppressing progesterone production), oral contraceptives, tamoxifen, antihistamines, prednisone, dapsone, thalidomide, azathioprine and danazol [7–9]. However, if these therapies fail, or cause intolerable side effects, a bilateral oophorectomy can be performed as a more durable solution [7].

## Case Presentation

A 34-year-old female with pronounced Cushingoid features presented to our Endocrinology clinic in 2018. She had been taking high dose oral prednisone for a diagnosis of severe autoimmune progesterone urticaria, refractory to bilateral oophorectomy.

**Fig. 1:**
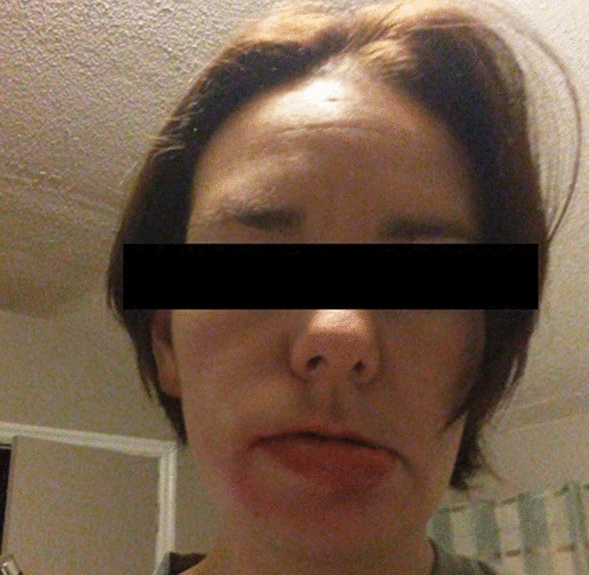
Acute angioedema—patient photograph, consent obtained

A raised pruritic macular erythematous rash initially appeared on her arms and legs in 2009, two to three days after the birth of her second child. This rash was associated with headaches, and diffuse angioedema of her hands, feet, lips, and eyelids (Fig. 1). The pruritic rash continued to occur in a cyclical fashion, appearing one to two weeks prior to each menses and resolving within a few days of menstruation onset. Six months after the post-partum period, in tandem with the return of her menstrual cycles, her symptoms quickly progressed from cyclic urticaria to systemic symptoms such as dyspnea. Her flare-ups also coincided with nausea, dizziness, and abdominal cramping. All symptoms continued to follow the identical luteal phase cyclical onset of presentation.

For several years, she was treated with numerous medications, including first and second generation H1 and H2 histamine antagonists, tricyclic antidepressants, calcineurin inhibitor immunosuppressant therapy and psoralen and ultraviolet A (PUVA) therapy. These treatments were all unsuccessful in improving her symptoms. She was eventually started on oral prednisone in doses of up to 100 mg daily. Symptomatic improvement was achieved with corticosteroid use, but pre-menstrual flare-ups would still occur quite often. After three years of persistent symptoms, she was referred to an Immunologist who established a likely diagnosis of autoimmune progesterone dermatitis with potential catamenial anaphylaxis. This was confirmed using a progesterone skin test. Her serum tryptase at baseline was low at 2.8 Ug/L (3.8–11.4 Ug/L).

**Fig. 2:**
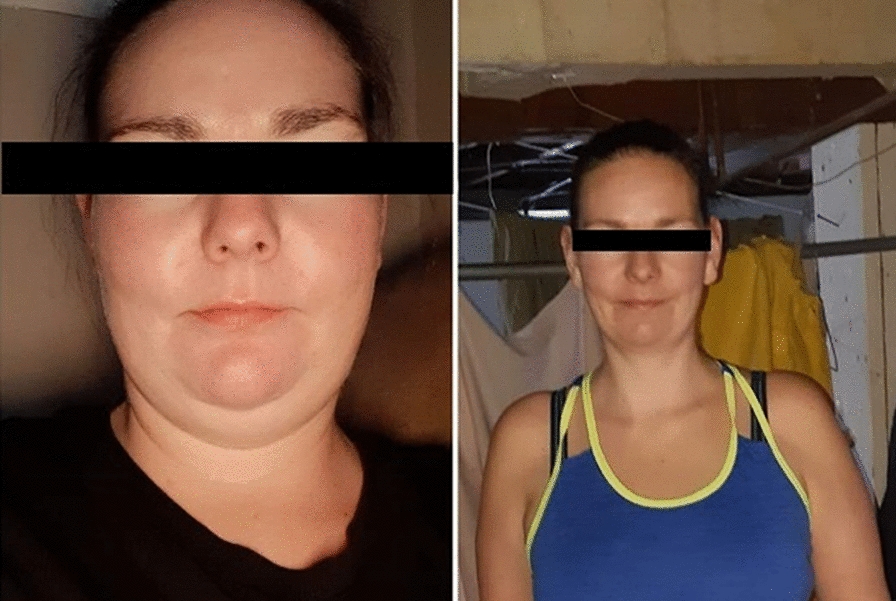
Iatrogenic cushing’s syndrome (left panel—at the time of Xolair initiation), followed by drastic weight loss off prednisone (right panel—approximately one year steroid free), patient photograph, consent obtained

She was then started on a trial of a low-dose oral contraceptive pill, which contained 0.02 mg ethinyl estradiol and 0.1 mg of levonorgestrel, in an attempt to be desensitized to progesterone. High dose prednisone was prescribed for flare-ups. This was an attempt to wean her off prednisone. After 6 months, there was still no improvement in her symptoms. The reintroduction of antihistamine therapy, intensified up to quadruple therapy, as well as a trial of cyclosporine therapy also did not allow for adequate prednisone taper. Gonadotropin-releasing hormone (GnRH) therapy was also recommended. However, the patient declined additional medical therapies. Due to the long-term use of high-dose prednisone, she developed Cushingoid features including moon face, violaceous striations, alopecia, and central adiposity **(**Fig. 2—left panel).

Due to the progressive side effects of prednisone as well as persistent symptoms, she elected to undergo a bilateral oophorectomy at age 34 in June 2018. There was short term relief of symptoms for approximately three weeks. Unfortunately, she experienced another flare-up of urticaria with angioedema which did not subside until treatment with prednisone 50 mg daily was reinstituted.

Unfortunately, following oophorectomy, her condition was further complicated by vasomotor menopausal symptoms, progressive iatrogenic Cushing’s syndrome and unresolved APD.

## Discussion

### Case Dilemma #1: Refractory APD Following Bilateral Ophorectomy

In our case, the patient had elected to proceed with a bilateral oophorectomy after three years of unresolving symptoms despite various treatment modalities. Bilateral oophorectomy for unresolving APD has been used 19 times and was found to be successful in all reported cases [1, 10–14]. However, in our case, the patient continued to have breakthrough episodes of generalized urticaria and peripheral swelling. These symptoms were in line with what she was experiencing before her bilateral oophorectomy. The differential diagnosis may also include chronic spontaneous urticaria and angioedema post-oophorectomy. Although she has not been retested using a medroxyprogesterone skin test since her procedure, the original ADP diagnosis was based on a positive skin test and strong clinical features in keeping with this diagnosis. Furthermore, a secondary workup for urticaria was also negative ruling out additional inflammatory, vasculitic, rheumatologic, endocrine and immunological conditions. Therefore, we believe that it would be unlikely that the underlying etiology of her symptoms would change with surgery and we presume an adrenal source of progesterone production post-oophorectomy. To our knowledge, this is the first case where a bilateral oophorectomy procedure may have failed to resolve symptoms of APD. However, one potential publication bias could indeed be a lack of reporting of non-responsive cases.

Three months following bilateral oophorectomy, she presented to our Endocrine clinic for an initial consultation for Cushing syndrome while being treated with 50 mg of daily prednisone. In search of a prednisone sparing treatment for her APD, we initiated Omalizumab (Xolair) monoclonal antibody therapy. IgE antibodies bind to the FcεRI region on the surface of the high-affinity IgE receptors, allowing for greater stabilization of the receptors. Cross-linking of IgE or stimulation of the high-affinity IgE receptor on mast cells leads to release of mast cell mediators including histamine. Omalizumab works by decreasing the amount of free IgE antibodies in circulation leading to a downregulation of the high-affinity IgE receptors on mast cells, thereby inhibiting the release of mast cell mediators [15–17]. Subcutaneous injections of Omalizumab 300mg allowed the patient to eliminate the requirement of daily oral prednisone over the course of six months with a slow taper. Most significantly, her ADP flare-ups have resolved. This is consistent with two other case reports which have shown improvement in symptoms of APD with Omalizumab [18, 19]. This is the third case for which Omalizumab was used for treatment for APD to our knowledge. We continue to attempt Omalizumab dose and frequency reduction, which she has not tolerated so far.

### Case Dilemma #2: Complications Of Early Menopause Secondary To A Bilateral Ophorectomy Procedure

Following her bilateral oophorectomy as well as chronic prednisone use, our patient was at risk for symptomatic early menopause and premature osteoporosis. As she had not responded to progesterone desensitization in the past, typical hormone replacement therapy, consisting of estrogen and progesterone, could not be considered. In addition, her uterus remained in situ, therefore treatment with unopposed estrogen would have put her at risk for endometrial hyperplasia and subsequent malignancy [20]. In hindsight, consideration of hysterectomy in addition to the bilateral oophorectomy would have negated the requirement of progesterone and allow for estradiol monotherapy. A hysterectomy may still be considered for the future. Unfortunately, estradiol monotherapy was not possible for our patient. As a result, we started the patient on the combination of conjugated estrogens with bazedoxifene. Several large Phase 3 trials have shown this combination to be effective in reducing vasomotor menopausal symptoms, as well as maintaining bone health and reducing the risk of endometrial hyperplasia for post-menopausal women between 40 and 65 years of age with an intact uterus [21–23]. Initiation of this treatment resulted in immediate vasomotor symptom relief.

### Case Dilemma #3: The Consequences Of Long-Term Use Of High Dose Oral Prednisone

Our patient was on sporadic prednisone since 2008. Following her bilateral oophorectomy in June 2018, she was taking prednisone 50 mg daily for three months prior to our initial consultation. Despite APD symptom improvement, long-term glucocorticoid usage has a myriad of possible adverse side effects, including lowered bone mass, myopathy, hyperglycemia, Cushing’s syndrome, and iatrogenic adrenal insufficiency [24]. In fact, our patient suffered from most of these complications.

As expected, she developed significant Cushingoid features including a significant weight gain of 70 lbs over four years, depression, proximal muscle wasting, moon face, easy bruising and dorsoclavicular adiposity. She has regained muscle mass and lost 35 lbs since prednisone discontinuation with resolution of the Cushingoid features (Fig. 2—right panel).

Our patient’s chronic use of glucocorticoid coupled with her bilateral oophorectomy puts her at significant risk for low bone mass and early development of osteoporosis. In August 2018, her bone mineral density showed lumbar spine osteopenia (T-Score of Lumbar Spine: − 1.1, Z-Score of Lumbar Spine: − 2.0, T-Score of Femoral Neck: − 0.8, Z-Score of Femoral Neck: − 1.1, T-Score of Total Hip: − 0.5 and Z-Score of Total Hip: − 0.9) and she was started on Calcium, Vitamin D supplements, as well as conjugated estrogens with bazedoxifene.

Furthermore, it was postulated that she had an episode of prednisone-induced adrenal insufficiency following a quick taper of daily oral prednisone, resulting in an episode of severe atypical chest pain, muscle aches, and nausea. Along with these symptoms, her fasting AM cortisol level was 86 mmol/L (135–537 mmol/L) roughly 48 hour after prednisone discontinuation. Due to iatrogenic adrenal suppression, the steroid taper occurred very slowly over the course of 6 months. Since this time, the patient has remained off steroids and is asymptomatic with cortisol levels within physiological range.

Throughout the many years of glucocorticoid therapy, she began to develop diffuse indeterminate bone pain and arthralgias which persist to this day. Multiple imaging modalities were ordered, which revealed no bone abnormalities. Muscle and joint pain have been reported with conjugated estrogens with bazedoxifene [25], however transient discontinuation of conjugated estrogens with bazedoxifene did not improve symptoms. Omalizumab serum sickness and other possible rheumatological conditions were also ruled out by her Immunologist. The cause of the symptoms remains inconclusive, but they are improving with physiotherapy.

## Conclusions

This patient case depicts a complex case of APD which began following a pregnancy. In contrast to the 19 previously documented cases, a bilateral oophorectomy did not lead to resolution of symptoms. This unique case identifies an additional steroid sparing therapeutic option, omalizumab, that can be considered for the treatment of APD. Given the apparent effectiveness of Omalizumab, consideration to using this drug should be given before proceeding on to bilateral oophorectomy surgery for APD.

This case also highlights the unique role of conjugated estrogens with bazedoxifene for vasomotor menopause treatment in patients with APD post bilateral oophorectomy if the uterus remains intact. Again, perhaps hysterectomy should be considered in future cases as it may allow estrogen monotherapy.

Lastly, this case report demonstrates the complications of long-term steroid therapy as well as the challenges encountered in discontinuing their use. Omalizumab therapy allowed for steroid discontinuation and the conjugated estrogens with bazedoxifene allowed for bone protection against steroid induced bone demineralization.

## Data Availability

The data use and analyzed during the current study are available from the corresponding author on reasonable request.

## Abbreviations

APD: Autoimmune progesterone dermatitis
IgE: Immunoglobulin E
GnRH: Gonadotropin releasing hormone
PUVA: Psoralen and ultraviolet A

## References

[1] Nguyen T, Ahmed AR (2016). Autoimmune progesterone dermatitis: update and insights. Autoimmun Rev.

[2] Mbonile L (2016). Autoimmune progesterone dermatitis: case report with history of urticaria, petechiae and palpable pinpoint purpura triggered by medical abortion. S Afr Med J.

[3] Snyder JL, Krishnaswamy G (2003). Autoimmune progesterone dermatitis and its manifestation as anaphylaxis: a case report and literature review. Ann Allergy Asthma Immunol.

[4] Prieto-Garcia A, Sloane DE, Gargiulo AR, Feldweg AM, Castells M (2011). Autoimmune progesterone dermatitis: clinical presentation and management with progesterone desensitization for successful in vitro fertilization. Fertilit sterilit.

[5] Camões S, Sampaio J, Rocha J, Tiago P, Lopes C (2017). Autoimmune progesterone dermatitis: case report of an unexpected treatment reaction. Australas J Dermatol.

[6] Solomon M, Itsekson AM, Lev-Sagie A (2013). Autoimmune progesterone dermatitis. Curr Dermatol Rep.

[7] Medeiros S, Rodrigues-Alves R, Costa M, Afonso A, Rodrigues A, Cardoso J (2010). Autoimmune progesterone dermatitis: treatment with oophorectomy. Clin Exp Dermatol.

[8] Zhang M, Tang X, Zhou H, Liao Q, Han J (2020). Case of autoimmune progesterone dermatitis presenting as necrotic migratory erythema successfully controlled by danazol. J Dermatol.

[9] Le K, Wood G (2011). A case of autoimmune progesterone dermatitis diagnosed by progesterone pessary. Australas J Dermatol.

[10] Whitt W, Stiegler JD, Richardson CT (2020). Autoimmune progesterone dermatitis mimicking facial erythromelalgia successfully treated with hysterectomy and bilateral salpingo-oophorectomy. JAAD Case Rep.

[11] Galán-Gutierrez M, Gomez-Arias PJ, Rodenas-Herranz T, Ruiz-Villaverde R (2020). Autoimmune progesterone dermatitis: successful outcome with bilateral salpingo-oophorectomy. Dermatol Ther.

[12] Drayer SM, Laufer LR, Farrell ME (2017). Autoimmune progesterone dermatitis presenting as Stevens–Johnson syndrome. Obstet Gynecol.

[13] DeRosa I, Bender B, Centilli M (2018). Autoimmune progesterone dermatitis. Cutis.

[14] Grunnet KM, Powell KS, Miller IA, Davis LS (2017). Autoimmune progesterone dermatitis manifesting as mucosal erythema multiforme in the setting of HIV infection. JAAD Case Rep.

[15] Spector SL, Tan RA (2007). Effect of omalizumab on patients with chronic urticaria. Ann Allergy Asthma Immunol.

[16] Chang TW, Chen C, Lin CJ, Metz M, Church MK, Maurer M (2015). The potential pharmacologic mechanisms of omalizumab in patients with chronic spontaneous urticaria. J Allergy Clin Immunol.

[17] Potaczek DP, Kabesch M (2012). Current concepts of IgE regulation and impact of genetic determinants. Clin Exp Allergy.

[18] Heffler E, Fichera S, Nicolosi G, Crimi N. Anaphylaxis due to progesterone hypersensitivity successfully treated with omalizumab. J Allergy Clin Immunol Pract. 2017;5(3):852–54. 10.1016/j.jaip.2017.01.003 28258855

[19] Gadoury-Levesque V, Bernstein J (2018). A CASE OF REFRACTORY RECURRENT CYCLICAL ANGIOEDEMA AND URTICARIA SUCCESSFULLY TREATED WITH MULTIPLE STEPS THERAPY. Ann Allergy Asthma Immunol.

[20] Gompel A (2020). Progesterone and endometrial cancer. Best Pract Res Clin Obstet Gynaecol.

[21] Pinkerton JV, Utian WH, Constantine GD, Olivier S, Pickar JH (2009). Relief of vasomotor symptoms with the tissue-selective estrogen complex containing bazedoxifene/conjugated estrogens: a randomized, controlled trial. Menopause.

[22] Pinkerton JV, Harvey JA, Lindsay R, Pan K, Chines AA, Mirkin S, Archer DF, SMART-5 Investigators (2014). Effects of bazedoxifene/conjugated estrogens on the endometrium and bone: a randomized trial. J Clin Endocrinol Metab.

[23] Silverman SL, Christiansen C, Genant HK, Vukicevic S, Zanchetta JR, de Villiers TJ, Constantine GD, Chines AA (2008). Efficacy of bazedoxifene in reducing new vertebral fracture risk in postmenopausal women with osteoporosis: results from a 3-year, randomized, placebo-, and active-controlled clinical trial. J Bone Miner Res.

[24] Oray M, Abu Samra K, Ebrahimiadib N, Meese H, Foster CS (2016). Long-term side effects of glucocorticoids. Expert Opin Drug Saf.

[25] Chaplin S (2016). Duavive HRT: conjugated oestrogens with bazedoxifene. Prescriber.

